# Predicting football match outcomes: a multilayer perceptron neural network model based on technical statistics indicators of the FIFA world Cup

**DOI:** 10.3389/fspor.2025.1705198

**Published:** 2025-12-03

**Authors:** Yingling Luo, Tao Quan, Yongfeng Cao

**Affiliations:** College of Physical Education and Health Science, Chongqing Normal University, Chongqing, China

**Keywords:** match outcomes prediction, technical statistics indicators, neural network model, machine learning, FIFA world cup

## Abstract

This paper utilizes the strong non-linear approximation capability of a multilayer perceptron Neural Network to predict match outcomes based on Technical Statistics Indicators. Principal component analysis was applied to all the official data for dimensionality reduction and feature identification, resulting 22 technical statistics indicators. An architecture of a Multilayer Perceptron Neural Network with a 24-4-3 was constructed using SPSS. The results showed that the model achieved an overall prediction accuracy of 86.7%, the prediction accuracy for Draw is substantially lower than for the Win and Loss. The neural network model exhibited robust predictive performance. On this basis, five relevant topics were discussed, including model performance evaluation, relationship between TSI and match outcomes, discriminative power of TSI, impact of stage on prediction results and incorrect predictions of match. Thus, coaches can enhance the team's performance-oriented results under limited training resources by transforming the high-impact technical statistical indicators identified by the model into training priorities, thereby achieving data-driven scientific training management.

## 1 Introduction

With the continuous development of contemporary sports, competitive matches have become increasingly complex and intense, while post-match datasets have expanded both in dimensionality and volume. Traditional analytical methods are often inadequate to capture the nonlinear, dynamic, and interactive characteristics of modern football performance data, thereby posing greater challenges for performance analysis. However, the rapid advancement of artificial intelligence and big data technologies has made the prediction of athletes’ match performance a realistic and achievable objective. In professional sports, predictive models that integrate historical match data, players’ physiological indicators, and contextual environmental factors have emerged as powerful tools for tactical optimization and injury risk management. Consequently, predicting football match outcomes has evolved from an almost insurmountable challenge into a systematic and feasible analytical endeavor. Within this context, the application of machine learning in sports science demonstrates considerable potential. Various machine learning and data analysis techniques are being applied to the field of soccer (1). Originating from the field of artificial intelligence, machine learning has increasingly been adopted in sports research as an indispensable tool for outcome prediction, and it is now recognized as a significant frontier in the academic study of sports performance.

Extensive research has been conducted to evaluate the accuracy of predictive models in forecasting match results. For instance, a Bayesian dynamic generalized linear model has been developed to predict football match outcomes (2). Related studies have also introduce novel features such as momentum and fatigue as part of the model development process (3). An expert-constructed Bayesian Network (BN) was applied to matches played by Tottenham Hotspur Football Club between 1995 and 1997 (4). Artificial Neural Network (ANN) models were employed to forecast the outcomes of the 2006 FIFA World Cup, achieving an accuracy rate of 76.9% (5), while other ANN-based approaches reported accuracy rates as high as 85% (6). Comparative analyses of back propagation (BP) neural networks and multiple regression models demonstrated that BP networks yielded superior predictive performance (7). Although previous studies highlight the strong predictive capability of machine learning models in sports competitions, several gaps and unresolved issues remain. In particular, the selection of input features has often depended on researchers’ subjective judgment. Previous studies have often overlooked the underlying multicollinearity and dimensional overlap among input variables, which may compromise the stability of technical statistics indicators (TSI), this study introduces Principal Component Analysis (PCA) to identify the latent structure among TSI and to select representative original variables that capture the major performance dimensions. These structurally validated TSI are then used as inputs for a Multilayer Perceptron (MLP) model to perform nonlinear modeling and outcome prediction. This integrated approach combines the dimensional interpretability of PCA with the nonlinear learning capacity of MLP, thereby enhancing both predictive accuracy and model robustness while maintaining interpretability, offering a scientifically grounded and practically relevant framework for football match outcome prediction.

In addition to this, there has been limited attention given to variations across tournament stages, such as group vs. knockout phases. Thus, the analysis stratifies competition stages to assess the robustness and applicability of predictive TSI across different competitive contexts. Finally, there is also a separate analysis of matches where the model predicted incorrectly. This study aims to leverage the PCA and MLP model to extract critical determinants from a large and complex set of TSI, thereby examining its effectiveness across tournament stages. The quantitative insights generated by this model not only provide coaches with evidence-based guidance for prioritizing training content but also offer an objective foundation for tactical decision-making and strategic adjustments.

## 2 Stats and methods

### 2.1 Procedure

To investigate the predictive capability of MLP models in predicting match outcomes during the FIFA World Cup, this study followed a systematic methodology comprising several key steps. The study is carried out through the following steps:

Step 1, FIFA Official Stats of the 64 matches were collected into Excel, with each match including 44 TSI for the two teams. Additionally, the stage identifiers (groups = 1, knockout = 2) and outcome codes (Loss = 0, Draw = 1, Win = 3) were appended to the primary database in accordance with FIFA competition regulations. All data were obtained from official match reports published on FIFA official website. The reliability and validity of which have been widely recognized and verified (8, 9).

Step 2, TSI selection: the predictor variables need to be more accurate (10). First, preprocess these data through standardization. Subsequently, screen and determine 22 TSI using the method of PCA.

Step 3, A MLP neural network model was constructed to predict match outcomes by SPSS V30.0 (11), and the model performance as well as the incorrectly predicted results were analyzed.

### 2.2 Selection of TSI

An excessive number of input nodes can impair the neural network's convergence and stability, and may even lead to overfitting. Therefore, it is necessary to conduct factor analysis on the initially selected TSI to remove those with strong multicollinearity. Furthermore, the determinants of winning in soccer across different leagues are inherently complex, requiring consideration of multiple dimensions (12, 13). This step aims to eliminate highly correlated variables and reveal the multidimensional structure of the selected TSI through principal component analysis, thereby laying the foundation for the predictive performance of subsequent models. The suitability of the data for factor analysis was verified using PCA. The KMO value exceeded 0.6, and the significance level of Bartlett's test was less than 0.01, indicating sufficient correlations among the variables. Since both tests are satisfied, the selected TSI are suitable for factor analysis.

After performing principal component analysis using SPSS and applying a varimax rotation to the factor loadings, the rotated component matrix was obtained. Based on the magnitude and clustering of variable loadings, the 22 indicators were grouped into five thematic dimensions: Distribution & Attacking, Shooting & Goal Scoring, Discipline, Set Plays and Defending. The corresponding variables are as follows:X_1_(PC), X_2_(CC), X_3_(OR), X_4_(BP), X_5_(IC), X_6_(FTE), X_7_(CLB), X_8_(CDLB), X_9_(OT), X_10_(AS), X_11_(GS), X_12_(FA), X_13_(YC), X_14_(RC), X_15_(OS), X_16_(CK), X_17_(K), X_18_(PS), X_19_(GP), X_20_(GP), X_21_(FT), X_22_(PA). The classification and loadings of all technical Statistics Indicators are shown in Table 1.

**Table 1:** The classification and loadings of technical statistics indicators.

Categories.	Serial No.	Abbr.	Description	Loadings
Distribution& attacking	X_1_	PC	Passes completed	.934
	X_2_	CC	Crosses completed	.796
	X_3_	OR	Offers to receive	.936
	X_4_	BP	Ball possession	.878
	X_5_	IC	In contest	.487
	X_6_	FTE	Final third entries	.778
	X_7_	CLB	Completed line breaks	.836
	X_8_	CDLB	Completed defensive line breaks	.550
Shot & goal	X_9_	ST	Shot on target	.657
	X_10_	AS	Assists	.901
	X_11_	GS	Goals	.921
Discipline	X_12_	FA	Fouls against	.782
	X_13_	YC	Yellow cards	.681
	X_14_	RC	Red cards	.855
	X_15_	OS	Offsides	.851
Set plays	X_16_	CK	Corner kicks	.687
	X_17_	FK	Free kicks	.498
	X_18_	PS	Penalties scored	.811
Defending	X_19_	GP	Goal preventions	.637
	X_20_	GC	Goal conceded	.852
	X_21_	FT	Forced turnovers	−.385
	X_22_	PA	Pressing applied	−.653

### 2.3 Stats pre-processing

Considering that football performance indicators are inherently team-specific rather than match-pair dependent, the unit of analysis is defined at the team-match level, implying that there are two independent observations for each match. This approach effectively doubles the number of observations, thereby enhancing statistical robustness and improving model generalization.

Due to structural biases specific to football, considering that variations in match duration caused by different competition stages might affect the prediction results. Upon verification, five knockout-stage matches ended in a Draw within 90 min and therefore proceeded to extra time according to competition rules. Therefore, we have uniformly applied per-90 normalization procedure to these matches, as shown in the following formula: $X_{\mathrm{per}90}=\frac{X_{\mathrm{raw}}}{T_{\mathrm{match}}}\times 90$. Since ball possession and shared possession are expressed as percentages, they were not individually adjusted, as this normalization does not influence other TSI.

Finally, due to the inconsistent scales of different types of data, neural networks may tend to focus on features with larger values and ignore those with smaller values during training, leading to unbalanced learning and unstable model performance. Unnormalized data can cause numerical instability, especially when computing activation functions, which can reduce the model's generalization ability. Therefore, normalizing data is an important step. For all continuous TSI, this study employed the (X-Min)/(Max-Min) method to normalize them to the interval [0, 1]. Categorical variables (e.g., match stage, match outcome) were processed using dummy variable encoding and were excluded from numerical normalization. Examples of the data before and after normalization are shown in Table 2.

**Table 2:** Examples of normalized data for selected teams.

Team	Before			After		
	FK	YC	CC	FK	YC	CC
QATAR	19	4	4	0.56	0.5	0.24
ECUADOR	17	2	4	0.48	0.25	0.24
ENGLAND	16	0	7	0.44	0.5	0.41
IRAN	10	2	1	0.20	0.25	0.06
SENEGAL	14	2	7	0.36	0.25	0.41
NETHERLANDS	14	1	8	0.36	0.13	0.47

### 2.4 Neural network model of MLP

#### 2.4.1 The MLP neurons selection

Neural networks are artificially constructed nonlinear dynamic systems inspired by the human brain's neural structure and recognition mechanisms. They typically consist of an input layer, one or more hidden layers, and an output layer. Each layer containing a different number of neurons and distinct activation functions between layers. The random selection of the number of hidden neurons may lead to overfitting or underfitting problems (14).

**Figure 1:**
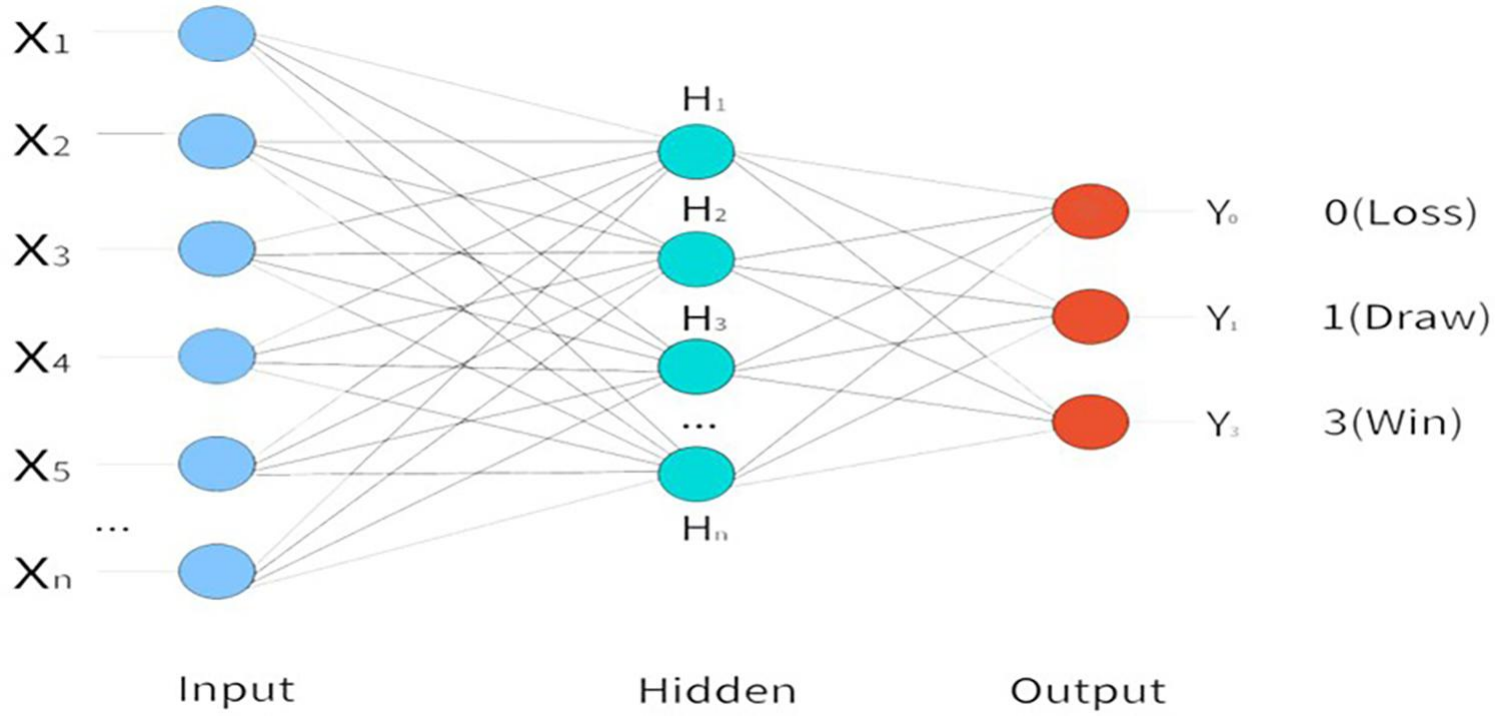
MLP neural nnetwork architecture.

Input Layer: The model utilizes 22 TSI as input variables, which serve as the input layer of the neural network. Output Layer: In the context of football match prediction, outcomes are typically classified into three categories-Win, Draw, and Loss. Accordingly, the output layer of the MLP neural network is configured with three neurons to represent these outcome classes. Hidden Layer: According to the universal approximation theorem, a feedforward neural network with a single hidden layer containing a sufficiently large number of neurons can approximate any continuous function on a compact subset of Rⁿ to an arbitrary degree of accuracy (15). This theoretical foundation supports the use of a single hidden layer in addressing complex nonlinear problems. However, the number of neurons in the hidden layer plays a critical role in determining the predictive performance of the network. An inappropriate number of neurons may result in either overfitting or underfitting. To address this, the present study adopts an automatic optimization strategy to determine the number of hidden units. Specifically, the modeling procedure constructs a network with one hidden layer and estimates the optimal number of neurons based on internal validation criteria. The model framework diagram is shown in Figure 1.

#### 2.4.2 Relevant parameters setting

The selection of activation functions determines the mathematical operations performed by individual neurons, influences the nonlinear representational capacity of the neural network, and directly affects its output characteristics and learning dynamics. An appropriate choice of activation function can enhance generalization performance and effectively mitigate the risk of overfitting.

The default parameter settings provided by SPSS were adopted. For case allocation, specifying (6, 2, 2) as the relative numbers for training, testing, and holdout samples corresponds to 60%, 20%, and 20%, respectively. According to the IBM SPSS Statistics Documentation, this method randomly assigns cases to each sample based on the specified relative proportions, ensuring mutual independence between the training and testing sets (16). Input Layer: As the input layer does not involve any nonlinear transformation, a linear activation function is generally employed. Hidden Layer: The tanh function is particularly effective in constructing nonlinear feature combinations and performs well with normalized input data. Output Layer: In football match prediction, the outcome can be categorized into one of three classes: Win, Draw, or Loss, thereby constituting a multi-class classification task. Accordingly, the soft-max activation function is employed in the output layer.

Several optimization algorithms are available for training MLPs, including backpropagation, conjugate gradient descent, and the Levenberg–Marquardt algorithm (17). In this study, the scaled conjugate gradient algorithm was selected. The initial hyperparameters were configured as follows: the lambda value is set to 0.0000005, the sigma value to 0.00005, the interval center to 0, and the interval offset to ±0.5. The learning algorithm iteratively updates the weights to minimize the discrepancy between the predicted outputs and the ground truth values (18). All the mathematical expression of the function and detailed parameterization is shown in Table 3.

**Table 3:** Relevant parameters of SPSS MLP neural network.

Category	Item		Setting
Partition dataset	Randomly assign		(6,2,2)
Architecture (Custom)	Hidden layer	Number of	1
		Number of units	Automatically compute
		Activation function	Hyperbolic tangent^a^
	Output layer	Number of	1
		Number of Units	3
		Activation function	Softmax^b^
Training	Type	Batch	
	Options	Initial Lambda	0.0000005
		Initial Sigma	0.00005
		Interval Center	0
		Interval Offset	±0.5

a *Y*(*c*) = tanh(*c*) = (*e^c^* − *e*^−*c*^)/(*e^c^* + *e*^−*c*^).

b *Y*(*c_k_*) = exp (*c_k_*)/*j*exp (*c_j_*).

## 3 Results

### 3.1 Information of network structure

Network Structure displays summary information about the neural network, including the dependent variables, number of input and output units, number of hidden layers and units, and activation functions. Network information is shown in Table 4.

**Table 4:** The information of network structure.

Network information		
Input layer	Factors	Stage
	Covariates	X_1_–X_22_
	Number of Units^a^	24
	Rescaling Method for Covariates	Normalized
Hidden layer(s)	Number of Hidden Layers	1
	Number of Units in Hidden Layer 1^a^	3
	Activation Function	Hyperbolic tangent
Output layer	Dependent Variables	Outcomes
	Number of Units	3
	Activation Function	Softmax
	Error Function	Cross-entropy

a Excluding the bias unit.

The Case Processing Summary shows that the data were split into 60.2% for training, 16.4% for testing and 23.4% for holdout, with no missing values, which meets standard machine-learning partitioning criteria. After running the program, the synaptic-weight diagram is displayed (figure omitted due to space limitations). An MLP with architecture 24-3-3 is constructed, the input layer consists of 22 TSI, 2 grouping variables (Groups and Knockout), and visually illustrates how the three layer are mapped to match outcomes probabilities through three tanh hidden nodes.

### 3.2 Model summary

Initially, the model was constructed without stage as a grouping variable, resulting in a decrease in overall predictive performance. Consequently, this study prioritizes findings derived from the model that incorporates stage as a predictor. After running the spss program, the model summary is shown in Table 5. The results indicate that the model attained a cross-entropy error of 18.526 and a misclassification rate of 5.2% on the training sample, 7.859 and 19.0% on the testing sample, and 13.3% on the testing sample. Training ceased after one consecutive iteration without improvement in test error, and the total training time was 0.01 s. The dependent variable is Outcomes, and all error metrics reported are calculated on the testing data. Overall, the model achieved approximately 86.7% accuracy on the test set with minimal training time, indicating a well-designed architecture and sufficient training.

**Table 5:** The summary of neural network model.

Model summary		
Training	Cross Entropy Error	18.526
	Percent Incorrect Predictions	5.2%
	Stopping Rule Used	1 consecutive step(s) with no decrease in error^a^
	Training Time	0:00:00.01
Testing	Cross Entropy Error	7.859
	Percent Incorrect Predictions	19.0%
Holdout	Percent Incorrect Predictions	13.3%
Dependent variable: Outcomes		

a Error computations are based on the testing sample.

### 3.3 Parameter estimates

The weights from the input layer to the hidden layer indicate that different TSI contribute differently to the three hidden neurons. The parameter estimates shown in Table 6 reveal that X_20_ (GC, −2.585), X_2_(CC, −0.748), X_3_(OR, −0.618), X_6_(FTE, −0.707), X_8_(CDLB, 0.704), X_19_(GP, −0.627) has a relatively high weight on H(1:1), X_9_(ST, 2.442), X_10_(AS, 3.682), X_11_(GS, 3.556), X_13_(YC, −1.302), X_18_(PS, 1.169), X_20_(GC, −3.517), X_21_(FT, 1.345) have a relatively high weight on H(1:2), X_1_(PC, −0.411), X_5_(IC, −0.744) has a relatively high weight on H(1:3).

**Table 6:** The parameter estimates of neural network.

Predictor		Predicted					
		Hidden Layer 1			Output Layer		
		H (1:1)	H (1:2)	H (1:3)	0	1	3
Input Layer	(Bias)	1.096	−.631	−1.329			
	[Groups]	.717	−.233	−.550			
	[Knockout]	1.294	−1.358	.071			
	X_1_(PC)	−.006	.366	−.411			
	X_2_(CC)	−.748	.092	−.235			
	X_3_(OR)	−.618	−.001	−.526			
	X_4_(BP)	−.589	−.739	−.278			
	X_5_(IC)	.441	−.716	−.744			
	X_6_(FTE)	−.707	.158	.038			
	X_7_(CLB)	−.162	.389	.362			
	X_8_(CDLB)	.704	.600	.352			
	X_9_(ST)	−.361	2.442	.407			
	X_10_(AS)	1.313	3.682	.281			
	X_11_(GS)	1.315	3.556	1.146			
	X_12_(FA)	−.305	−.077	−.384			
	X_13_(YC)	−.112	−1.032	.250			
	X_14_(RC)	.048	.958	−.168			
	X_15_(OS)	−.222	−.777	−.136			
	X_16_(CK)	.296	−.197	.104			
	X_17_(FK)	.027	.464	.127			
	X_18_(PS)	−.341	1.169	.274			
	X_19_(GP)	−.627	−.542	−.384			
	X_20_(GC)	−2.585	−3.517	−.103			
	X_21_(FT)	−.508	1.345	.246			
	X_22_(PA)	−.459	.434	.104			
Hidden Layer 1	(Bias)				1.466	.008	−1.168
	H (1:1)				−5.354	.945	4.325
	H (1:2)				−2.741	−.191	3.098
	H (1:3)				−.206	−1.505	.984

Connections from the hidden layer to the output layer reveal distinct pathways: H(1:1) have weights of −5.354 for Loss, 0.945 for Draw, and −1.168 for Win, reflecting a pathway more aligned with Loss predictions; H(1:2) have weights of −2.741 for Loss, −0.191 for Draw, and 3.095 for Win, corresponding to pathways associated with higher predicted probability of winning; H(1:3) has weights of −0.206 for Loss, −1.505 for Draw, and 0.984 for Win, indicating a pathway favoring Draw and lower alignment with Win.

Overall, the network reveals a nonlinear mapping between TSI and match outcomes. Predicted Loss, Win, and Draw are associated with different combinations of TSI, emphasizing that match outcome predictions in the MLP model depend on interactions among multiple indicators rather than any single variable alone. This highlights the suitability of MLP neural networks for capturing complex, nonlinear relationships in football performance data.

### 3.4 Model prediction results

The classification results are presented in confusion matrices for each categorical dependent variable, reported by partition and overall. Each matrix enumerates the number of correctly and incorrectly classified cases for each category of the dependent variable. Additionally, the overall classification accuracy, expressed as the percentage of correctly classified cases out of the total, is provided.

As shown in Table 7, the overall accuracy for the randomly assigned training set, testing set and holdout is 94.8%, 81% and 86.7%. Specifically, the prediction accuracy for outcome class 0 decreased from 42.9% to 30% whereas the accuracy for outcome class 1 increased from 19% to 20%, and that for outcome class 3 increased from 38.1% to 50%. Notably, the prediction accuracy for Draw is substantially lower than for the other two outcome categories, reinforcing the notion that Draw outcomes are inherently more difficult to predict accurately in football matches.

**Table 7:** Classification of the predicted outcomes.

Sample	Observed	Predicted			
		0	1	3	%
Training	0	32	0	0	100.00%
	1	1	17	2	85.00%
	3	0	1	24	96.00%
	Overall Percent	42.90%	23.40%	33.80%	94.80%
Testing	0	8	0	0	100.00%
	1	1	3	2	50.00%
	3	0	1	6	85.70%
	Overall Percent	42.90%	19.00%	38.10%	81.00%
Holdout	0	9	0	0	100.00%
	1	0	3	1	75.00%
	3	0	3	14	82.40%
	Overall Percent	30.00%	20.00%	50.00%	86.70%

### 3.5 Predicted by observed chart

**Figure 2:**
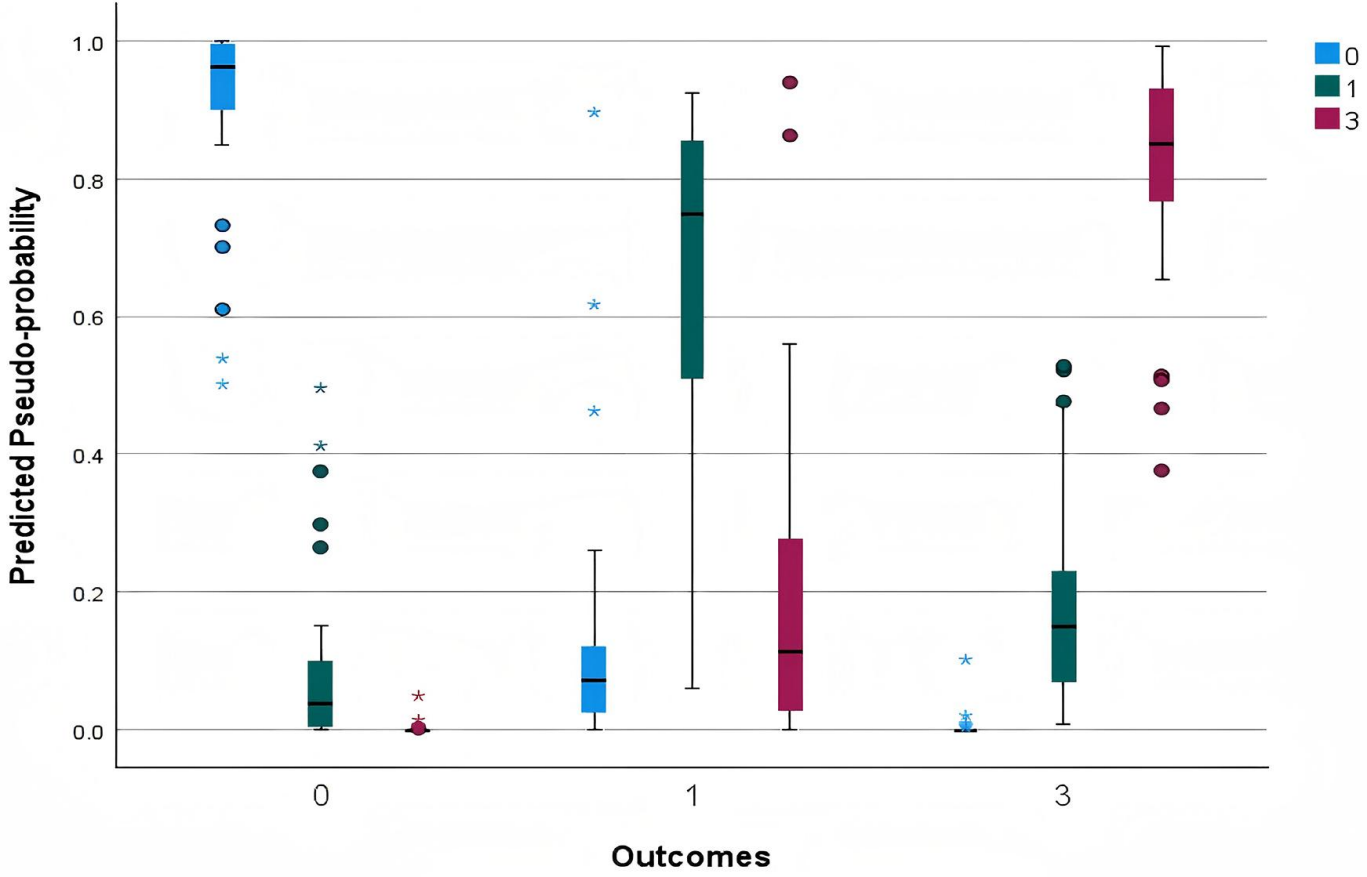
Predicted by observed chart.

According to Figure 2, the model's predicted probabilities for Win and Loss are predominantly concentrated near 1.0, indicating strong predictive performance for these categories. In contrast, the predictions for the Draw category are more dispersed. For matches that resulted in Draw, the range between the minimum and maximum predicted probabilities (excluding outliers) is notably wider, reflecting the model's lower confidence in accurately predicting Draw. Additionally, some Loss outcomes are misclassified with high predicted probabilities for either a Draw or a Win. Overall, the model demonstrates relatively robust performance in predicting Win and Loss but exhibits difficulty in accurately forecasting Draw. This observation aligns with previous findings that due to the inherent difficulty of scoring goals in football, Draw occurs frequently, making them challenging to predict. Typically, neural networks tend to effectively identify winners and losers but struggle with Draw. Notably, when Draw matches are excluded, prediction accuracy can improve substantially (5).

### 3.6 ROC curve

**Figure 3:**
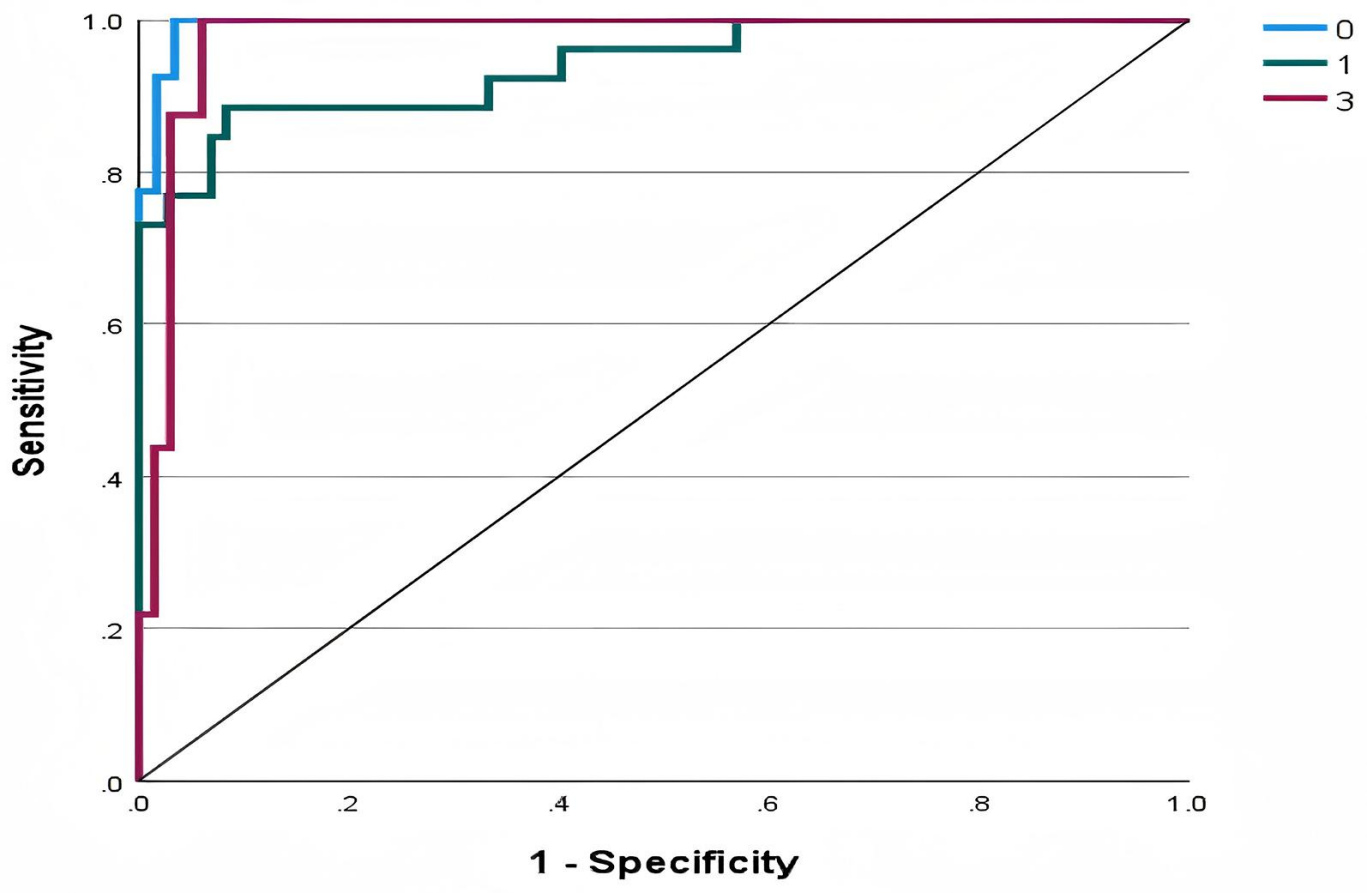
ROC curve of model.

The ROC curve is utilized to evaluate the trade-off between true positive rates and false positive rates. Figure 3 displays ROC curves corresponding to each category of the dependent variable. Specifically, for each categorical outcome, an individual ROC curve is plotted to illustrate the model's discriminatory ability. In this study, the areas under the curves (AUC) were 0.995 for Loss, 0.940 for Draw, and 0.976 for Win, indicating excellent predictive performance across all categories.

### 3.7 Cumulative gains chart

**Figure 4:**
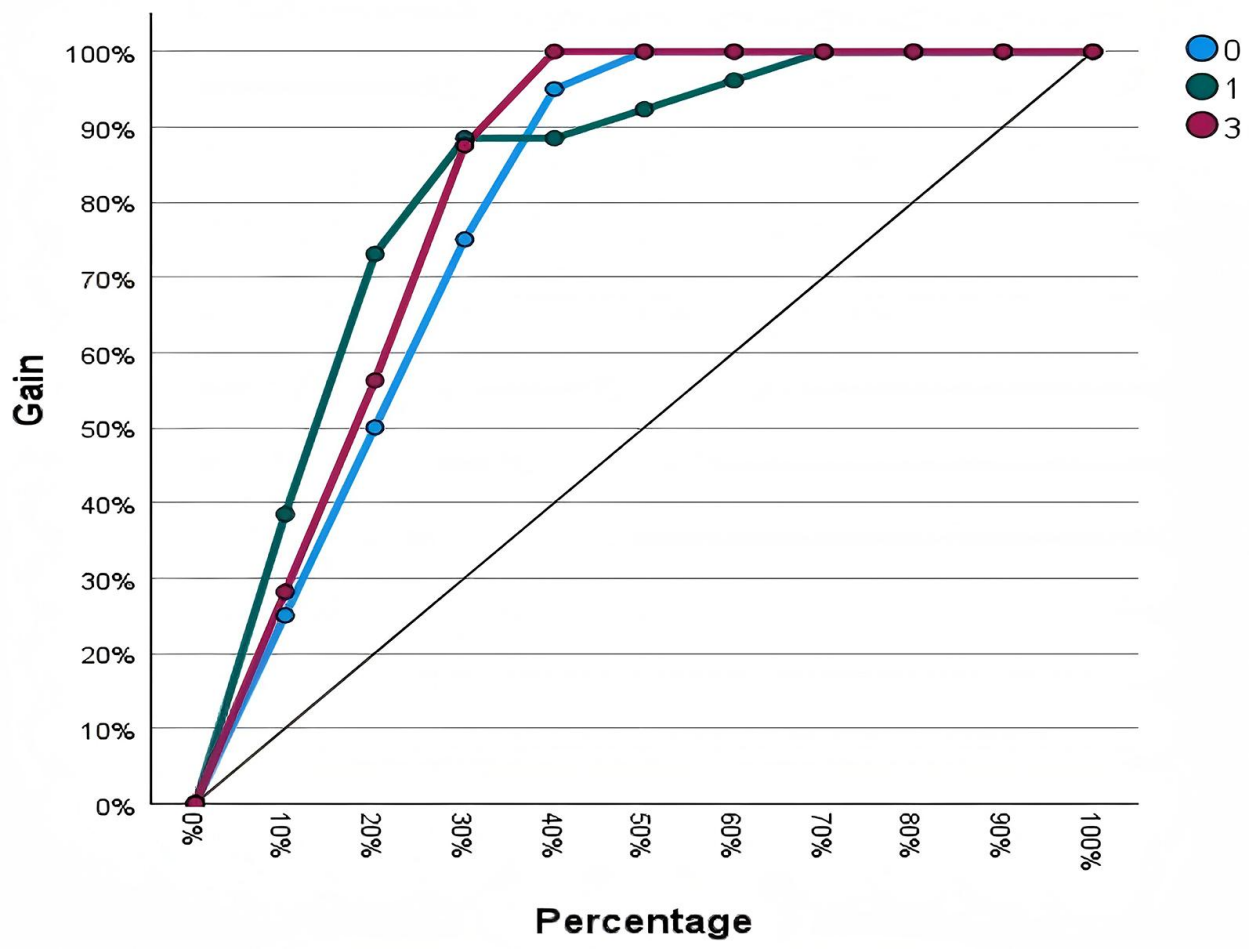
Cumulative gains chart of model.

The Gain Chart assesses the effectiveness of a classification model by comparing its predictive performance against a random baseline, thereby illustrating the model's capability to correctly identify target classes. Figure 4 displays the cumulative gains charts for each categorical outcome variable. All curves exceed the random baseline, demonstrating robust classification performance. Specifically, for match outcomes labeled as 0 and 3, approximately 95% of positive cases are captured within the top 40% of the sample, reflecting excellent predictive power. For the match outcome labeled as 1, the top 30% of the sample accounts for 90% of positive cases, indicating a highly efficient classification for this category.

### 3.8 Lift chart

**Figure 5:**
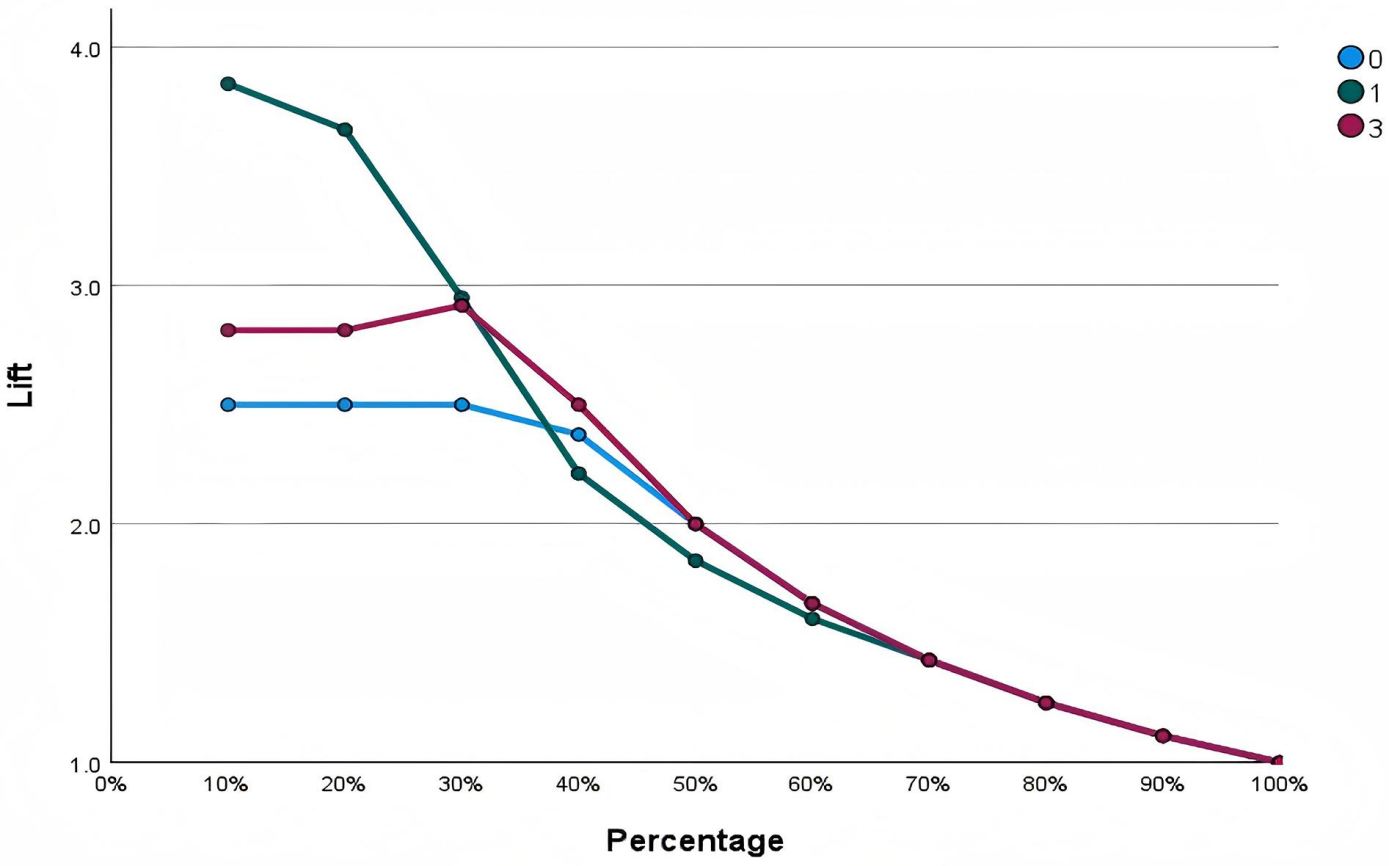
Lift chart of model.

Figure 5 displays the MLP neural network model's performance in predicting the three match outcomes. The *x*-axis represents the cumulative percentage of samples, and the *y*-axis represents the Lift value, indicating how much better the model performs compared to random guessing. Overall, the Lift values for all outcomes decrease as the sample percentage increases and converge toward 1 at 100%, suggesting that the model's discriminative ability is concentrated in high-confidence samples. Notably, the Draw outcome shows the highest Lift value (around 3.8) within the top 10% of samples, indicating strong predictive ability in a small subset of cases, whereas the Win and Loss curves remain more stable, reflecting a more balanced prediction performance across samples.

Although the Draw outcome exhibits the highest Lift value in the high-confidence range, this does not imply that the model performs best in predicting Draw overall. On the contrary, it is a poor predictor of Draw in terms of the overall performance of the model. Draw samples are typically much fewer than Win or Loss samples, leading the model to focus on optimizing predictions for the majority classes while having limited generalization ability for Draw. From a technical and tactical perspective, Draw often shows intermediate values between Win and Loss, making it difficult for the model to establish a clear decision boundary. A high Lift value indicates strong predictive confidence for a small subset of samples but does not reflect the model's overall classification stability. While the model can accurately identify a few typical Draw cases, it struggles to recognize Draw consistently across the full dataset, resulting in lower overall predictive performance for this category.

### 3.9 Independent Variable importance

**Figure 6:**
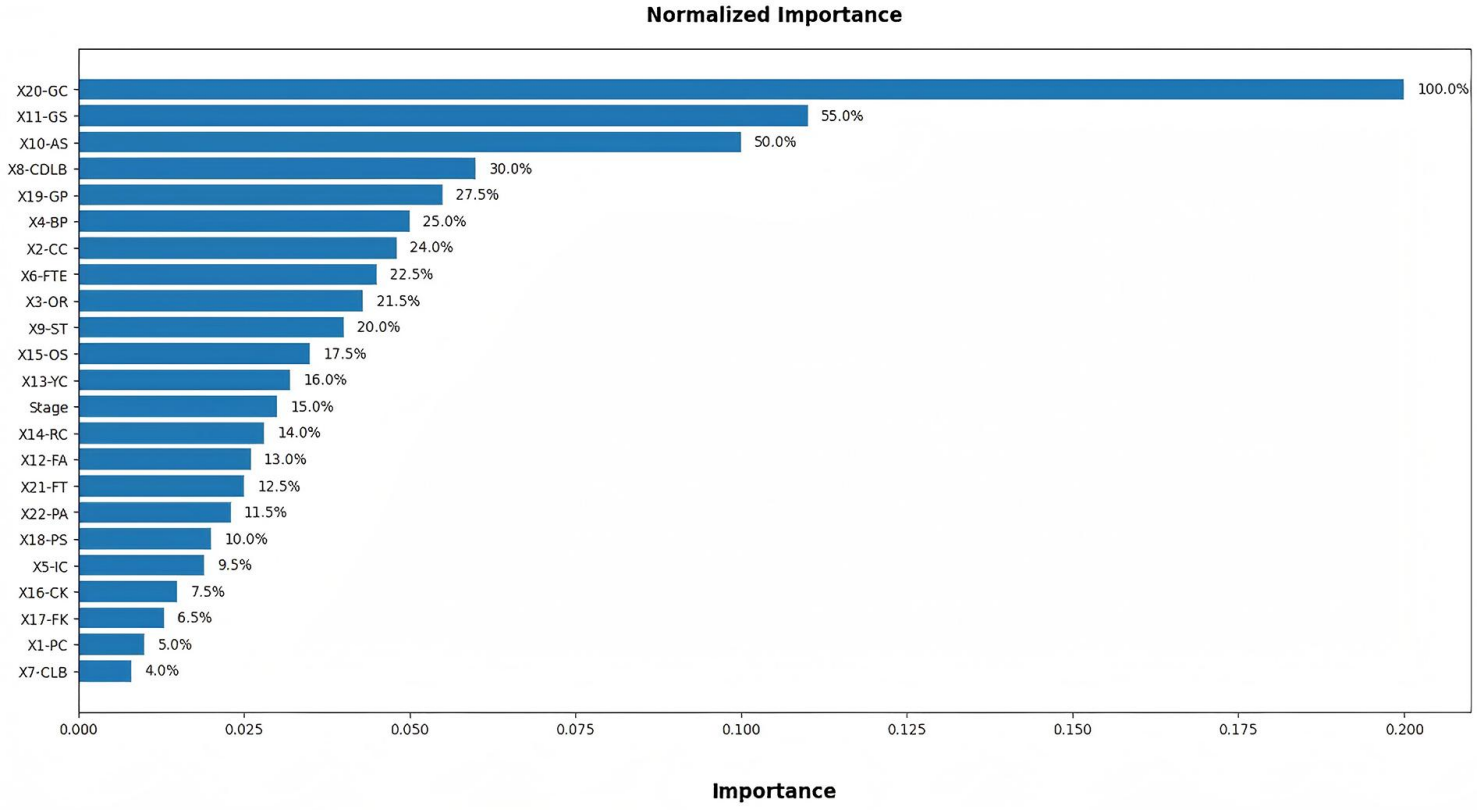
Independent Variable importance chart.

Figure 6 visually displays the variables ranked by their normalized importance, further emphasizing the relative significance of each performance indicator. The top three most impactful variables are GC at 100%, GS at 55%, and AS at 50%. A similar study revealed that “On Target”, “Shooting Opportunity”, and “Ball Progressions” were important (19). These TSI were significantly contributed to the model's predictive accuracy, highlighting their central role in determining match results and aligning with the fundamental objectives of football. In contrast, variables such as CLB at 4%, PC at 5%, FK at 6.5% are deemed less influential. Although penetrating the defensive line is widely recognized as a critical offensive action in modern football, this behavior is inherently context-dependent. Breakthrough attempts typically occur in specific tactical moments such as transition phases and counter-attacking situations. As such, their frequency does not necessarily reflect the overall offensive threat or scoring efficiency. Consequently, this variable contributes limited explanatory power when analyzed at a macro, whole-match level across large samples.

The number of completed passes, which is traditionally considered an indicator of technical stability and possession control. This primarily reflects a team's ball-retention capability rather than direct match-decisive impact. Modern match success is increasingly associated with vertical progression, speed of play, and quality of finishing actions, rather than the sheer accumulation of possession-related events. Thus, it is reasonable that this metric carries relatively weak predictive weight in the model. Freekick events accounted for a small proportion of total match actions and involve considerable event-specific randomness. Although set-pieces can be decisive in isolated or high-stakes matches, their low frequency and situational dependency dilute their statistical influence when modelling long-horizon competitive outcomes.

Collectively, these results do not negate the tactical relevance of the aforementioned variables. Rather, they underscore the importance of contextual and event-quality-based interpretation in football analytics. The findings suggest that predictive models of match outcomes should prioritize variables that directly influence scoring probability and defensive stability while incorporating contextual analysis to complement statistical modelling. This aligns with current analytical trends emphasizing micro-structural match-event significance over volumetric frequency indicators in high-performance football research.

The more technology advances, the more accurate the data becomes, and from the 2022 World Cup, relevant indicators were introduced as part of Enhanced Football Intelligence (EFI), such as In Contest, Line Breaks and Forced Turnovers. Specifically, a third category, “In Contest” was introduced to enhance the Possession (%) traditionally seen on TV screens. The possession state “In contest” is an accumulation of moments during the match when neither team are in controlled possession of the ball. This state is triggered by certain events that occur during the match. A Completed Line Break can go through, around or over a unit. “Completed Line breaks” also contains information on whether line-breaking passes, crosses or ball progressions have occurred inside or outside the opponent's team shape. Forced turnovers are a defensive metric awarded to the defending team. This indicator captures the moments when the attacking team loses position of the ball due to pressure being applied by the defending team. The higher the quality, intensity and number of player pressures, the higher the chance of the team in possession losing the ball. Teams and players will often be seen pressing or applying pressure in the opponents’ defensive third in order to force a turnover in possession close to the opponents’ goal, thereby increasing the opportunity of creating a goal-scoring opportunity. This type of mistake usually gives the opponent a chance to launch a fast counter-attack, and if it happens in the Defensive third, the threat skyrockets. From Figure 6, we can see that Completed Defensive Line Breaks both have relatively high discriminative power.

## 4 Discussion

### 4.1 Model performance evaluation

In order to evaluate the performance of the multilayer perceptron (MLP) model for predicting football match outcomes, we employed a series of standard classification metrics, including Accuracy, Precision, Recall, F1-score, Macro-F1, and Weighted-F1. Given that the SPSS neural network module does not directly output all detailed components, we manually extracted these values from the predicted and actual results to ensure precise calculation. To ensure precise and reproducible model evaluation, the predicted and actual match outcomes were first manually extracted from the system output. After confirming the correctness of the labels, the classification metrics, including confusion matrix, precision, recall, F1-score, and overall accuracy were calculated programmatically using the sklearn package in Python.

The model's evaluation involves the calculation of True Positives (TP), True Negatives (TN), False Positives (FP), and False Negatives (FN) shown in Table 8. Based on this, we calculated Accuracy (Acc), Precision (P), Recall (R), and F1 score, as shown in the Table 9.

**Table 8:** Confusion matrix.

Actual/Predicted	0	1	3
0	TP₀	FP₀ → 1	FP₀ → 3
1	FP₁ → 0	TP₁	FP₁ → 3
3	FP₃ → 0	FP₃ → 1	TP₃

TPi: True Positives for class *i* (matches correctly predicted as class *i*).

FPi → *j*: False Positives, matches of other classes incorrectly predicted as class *i*.

FNi: False Negatives for class *i* (matches of class i incorrectly predicted as other classes).

**Table 9:** Model performance evaluation.

Class	Precision	Recall	F1-score	Support
Loss (0)	0.961	1.000	0.980	49
Draw (1)	0.821	0.767	0.793	30
Win (3)	0.898	0.898	0.898	49
macro-avg		0.890		128
weighted-avg		0.905		128
Accuracy		0.906		

The model achieved strong overall performance (Accuracy, 0.906). It identified Loss (F1, 0.98) and Win outcomes (F1, 0.898) with high reliability. Performance for Draw predictions was relatively lower (F1, 0.793), indicating that matches ending in Draw were more challenging to classify, likely due to tactical and performance similarities between balanced contests and other match outcomes. The macro-avg (0.890) and weighted-avg (0.905) further confirm balanced predictive capability across categories.

### 4.2 Relationship between TSI and match outcomes

On the attacking side, Goals and Assists have consistently been identified as key explanatory TSI. Similar results have been reported by Kubayi et al. (2020), who found that teams that take more shots on target have more goal-scoring opportunities, which may increase their chances of winning matches (20–22). This is primarily because of their direct causal link with results. Goals are the ultimate determinant of the outcome, while Assists represent the immediately preceding action, reflecting a team's offensive organization and chance creation. Together, they are core measures of offensive efficiency and creativity. In football matches, not all passes or actions can significantly change the course of the game, whereas a Completed Defensive Line Breaks means that a player has successfully advanced the ball past the opponent's defensive line, breaking their defensive structure, and constitutes a high-value action. Previous research has shown that the winning team's defensive line breaks higher and has a correlation with goals scored, while running longer distances at higher speeds (23). Relative research also found that receptions behind the defensive line and completed defensive line breaks made a difference between winning and losing teams (24). Ball possession has a bearing on whether a game is won or lost. Ball possession indicates that a team controls the match, organizing attacks while limiting the opponent's influence, thereby increasing the likelihood of winning. Some studies have reported that in the final phases of tournaments, winning teams often recorded lower possession rates than losing teams (25). By contrast, analyses of the 2018 FIFA Men's World Cup revealed that successful teams surpassed their opponents in possession, passing accuracy, and time spent at sprinting speeds (26). Shots on target are a direct measure of scoring threat, with more shots on target corresponding to higher probabilities of scoring. These TSI capture a team's true effectiveness in attack–defense transitions, thereby typically carrying greater weight and significance in machine learning models. Kite et al. found that the team should adopt a more direct style of play. They should move the ball into a shooting position with fewer passes and ensure that more shots are on the target (27).

On the defending side, In contest, Goal Conceded, yellow cards and Offsides have a definite association with match results. “In contest” as Enhanced Football Intelligence (EFI) was introduced. High-intensity and effective contests not only facilitate ball recovery and disrupt the opponent's attacking rhythm but also enable pressing in key areas to create counterattacking or progression opportunities. Goals Conceded is one of the most direct and critical indicators influencing football match outcomes. Yellow cards, while not reducing player count, indicate disciplinary or defensive issues. A high frequency of yellow cards often forces players to adopt more conservative tactical behaviors, limiting offensive freedom and defensive flexibility, and indirectly reducing the probability of winning. Previous studies have confirmed this pattern, showing that red cards drastically reduce the chance of winning, while even yellow cards exert a negative influence by weakening defensive stability, particularly for away teams in tightly contested matches (28–30). Offsides reflect insufficient coordination in attacking movements, with frequent offsides indicating that offensive plays are effectively disrupted by the opponent's defensive line, leading to reduced attacking efficiency and lower likelihood of favorable match results. Collectively, these indicators capture a team's passive state, tactical limitations, and overall disadvantage, accounting for their significant negative association with Win.

### 4.3 Discriminative power of TSI

In terms of predictive performance, different TSI exhibit varying levels of importance. Variables such as Goals, Conceded, Assists, Completed Defensive Line Breaks, Goal Preventions, Possession and Crosses Completed exhibit higher importance in predicting match outcomes due to their strong causal relationships with the target variable. Goals, Conceded and Goal Preventions directly determine the match outcome and thus possess the highest signal strength, effectively reducing model entropy. Assists function as proximal precursors to scoring events, providing high predictive sensitivity, while ball possession and Completed Defensive Line Breaks encapsulate a team's structural dominance, reflecting continuity of attack, spatial control, and tempo regulation. Consequently, these TSI carry substantial information gain, which is inherently prioritized by the model during the feature-weighting process. Set pieces, especially free kicks also serve as important predictors, aligning with the logic of the game in which scoring is the ultimate objective. Empirical evidence highlights that winning teams consistently outperform losing teams in all set-piece variables, especially in set-piece attacks, set-piece shots, and corners (31).

Conversely, TSI such as Completed Line Breaks, Passes Completed, Free Kicks, Corner Kicks show lower importance because their relationships with match outcomes are indirect and context-dependent. The higher predictive importance of Completed Defensive Line Breaks is higher compared with Completed Line Breaks. Breaking the defensive line typically generates immediate goal-threat scenarios, such as entering spaces behind defenders or creating numerical superiority near the penalty area, which is more directly associated with shot creation and scoring probability. Completed Line Breaks represent broader circulation and territorial gain with weaker direct linkage to match-decisive actions, resulting in lower importance within the predictive model. Passes Completed primarily represent possession maintenance rather than direct offensive threat creation. Higher pass completion does not guarantee victory unless accompanied by forward-progressive and high-threat actions. Existing research has shown that teams with a higher number of passes and more frequent defensive line breaks are often required to engage in greater running activity (32). Thus, mere completion counts contribute limited predictive information once other quality-related metrics such as expected threat or xG are considered. Free kicks and Corner kicks are low-frequency, high-variance events. Their impact depends strongly on moment-specific execution quality, tactical design, and opponent defensive organization, which are not fully captured by simple event counts. Therefore, these variables exhibit weak statistical relevance in global predictive models.

This pattern indicates that machine learning models inherently prioritize outcome-oriented variables over those describing process-oriented or situational behaviors. Therefore, in this context, it is important for coaches to monitor the overall team performance using the high weighted metrics identified by the model while also focusing on the performance of the low weighted process metrics in specific contexts, as the contextual values that have a key impact on game dynamics and player decision making may be diluted in the global model training.

### 4.4 Impact of stage on predicted results

In football matches, stage usually refers to the phase division of the tournament, including groups and knockout. As a grouping variable, stage directly influences the match outcome (33). Comparison of Predicted Results of Stage vs. Non-Stage is shown in Table 10.

**Table 10:** Comparison of predicted results of stage vs. non-stage.

Observed	0		1		3		%	
	Non-stage	Stage	Non-stage	Stage	Non-stage	Stage	Non-stage	Stage
0	4	9	1	0	0	0	80.0%	100.0%
1	2	0	5	3	1	1	65.5%	75.0%
3	0	0	0	3	7	14	100.0%	82.4%
Overall	30.0%	30.0%	30.0%	20.0%	40.0%	50.0%	80.0%	86.7%

The staged model yields both higher and more stable accuracy than its non-stage counterpart, with no indication of increased overfitting, clear evidence that the grouping strategy successfully steers the network toward a superior decision boundary. Conservative by design, the models seldom misclassify instances once they commit to “Loss” and “Win”; the majority of errors are confined to cases where the observed label is “Draw”. By incorporating stage as a grouping variable, overall accuracy rises by +6.7%, from 80% to 86.7%, entirely through improved capture of the minority classes without any degradation on the majority class.

Adding stage as a grouping variable enhances the separability of extreme outcomes (Loss and Win) by leveraging the tactically divergent risk profiles that characterize group-stage vs. knockout-phase contests. It reallocates ambiguous instances away from the Draw class, aligning with the empirical rarity of drawn matches in knockout tournaments. Consequently, stage serves as a low-cost yet highly informative contextual feature that should be retained whenever the utility function prioritizes well-calibrated probabilities over raw predictive accuracy. It increasing separability of the extreme classes (Loss and Win) by exploiting the real-world tactical difference between group and knockout phases, re-allocates uncertain cases away from the Draw, reflecting the empirical fact that Draw are rarer in knockout football.

### 4.5 Incorrect predictions of match

The model is based on TSI for match outcome forecasting, it has achieved a prediction accuracy of 86.7%, demonstrating a certain level of stability and good performance. However, the model's prediction of Draw needs improvement. In the original SPSS database, the teams with incorrect predictions involved were identified through filtering and verification of the pseudo-probability for Loss, Draw, and Win. The incorrect prediction results involved are shown in Table 11.

**Table 11:** The incorrect prediction of match.

Match	Team	Match result	Predicted value	Pseudo-p 0	Pseudo-p 1	Pseudo-p 3
5	ARGENTINA	0	0	0.868	0.125	0.007
	SAUDI ARABIA	3	1	0.006	0.528	0.466
15	PORTUGAL	3	1	0.102	0.522	0.376
	GHANA	0	0	0.513	0.442	0.045
25	JAPAN	0	0	0.948	0.052	0
	COSTA RICA	3	1	0.003	0.57	0.427
32	PORTUGAL	3	1	0.06	0.556	0.384
	URUGUAY	0	0	0.991	0.009	0
62	FRANCE	3	1	0.006	0.705	0.29
	MOROCCO	0	0	0.823	0.177	0
28	SPAIN	1	3	0.008	0.459	0.533
	GERMANY	1	3	0.009	0.431	0.56
29	CAMEROON	1	3	0	0.137	0.862
	SERBIA	1	1	0.09	0.573	0.336
55	MOROCCO	1	1	0.006	0.732	0.261
	SPAIN	1	3	0	0.061	0.939
58	NETHERLAND	1	1	0.125	0.863	0.012
	ARGENTINA	1	3	0.001	0.399	0.6
57	CROATIA	1	0	0.617	0.381	0.002
	BRAZIL	1	1	0.026	0.917	0.057
64	ARGENTINA	1	0	0.896	0.104	0
	FRANCE	1	1	0.009	0.85	0.141

A total of 12 incorrect predictions involved 11 matches and 10 teams (Portugal twice, Argentina twice), Among, including 5 Wins predicted as Draws, 5 Draws predicted as Wins, and 2 Draws predicted as Loss. The detailed list is as follows:

1. Win → Draw (5 cases): Match 5 (Saudi Arabia), Match 15 (Portugal), Match 25 (Costa Rica), Match 32 (Portugal), Match 62 (France). Across these fixtures of the model's pseudo-probabilities for Win and Draw differ only marginally, making the final classification highly sensitive to small logit fluctuations. For instance, Saudi Arabia's pseudo-probabilities stood at 0.466 for Win and 0.528 for Draw, a marginal difference of 0.062 that tipped the prediction from Win to Draw.
2. Draw → Win (5 cases): Match 28 (Spain vs. Germany), Match 29 (Cameroon), Match 55 (Spain), Match 58 (Argentina). Results likewise show the model's pseudo-probabilities for Draw and Win differing only marginally. In Spain's case, for example, the output was 0.459 for Draw vs. 0.533 for Win, probabilities stood at 0.459 for Draw and 0.533 for Win, a slender gap of just 0.074 that tipped the prediction from Draw to Win.
3. Draw → Loss (2 cases): Match 57 (Croatia), Match 64 (Argentina). In these two matches, the model's pseudo-probabilities for Loss were 0.617 for Croatia and 0.896 for Argentina—both indicating a clear and strong prediction toward a Loss outcome. By reviewing the video review, it was found that a team's prediction is related to some key behavioral events on the field, such as the conversion rate of shoot, the performance of goalkeeper and superstars, the penalty decisions of referees, and the impact of offside, of course, this would not is inseparable from the intervention of high-tech equipment such as VAR(Video Assistant Referee) and SAOT(Semi-Automated Offside Technology).

In short, football is a game of attacking and defensive balance, fully in line with the performance-determinants framework of sports-training science, in which a team's competitive level is continually affected by the performance of the opponent's actions (34). Under the Laws of the Game, any single match involves two teams, if one is predicted to Win, the other must lose; if the forecast is a Draw, both are Draw, and neither Win nor Loss can appear. In football, a side can dominate every metric yet still fail to secure victory, an enduring reminder of the sport's unpredictability and enduring charm. This deterministic, mutually exclusive structure is presently ignored by neural-network classifiers and should be explicitly embedded in future architectures to ensure logically coherent predictions.

## 5 Conclusions

The 22 technical statistics indicators were categorized into five thematic dimensions: Distribution & Attacking, Shooting & Goal Scoring, Discipline, Set Plays and Defending. An architecture of MLP Neural Network with a 24-4-3 was constructed. The findings indicate that the model attained a notably high level of accuracy in predicting the football outcomes, achieving an overall prediction rate of 86.7%.

The present study also revealed a distinction between indicators showing relationship with match outcomes and those contributing most to the discriminative power of the MLP model. Indicators such as Goals, Assists, Completed Defensive Line Breaks, Ball Possession, In Contest, Goal Conceded, Yellow Cards, and Offsides demonstrated univariate associations with match results. However, when evaluated within the MLP framework, additional features, including Completed Line Breaks, Passes Completed, Free Kicks, and Corner Kicks—were found to exhibit substantial importance for outcome prediction. This discrepancy may arise from the inherent nonlinear and interactive mechanisms of MLP. The model captures complex feature interdependencies that enhance predictive performance even for variables with limited individual correlation. Overall, this method demonstrates good application effects in the prediction of football match results based on technical statistics indicators. The staged model yields both higher and more stable accuracy than its non-stage counterpart, with no indication of increased over-fitting—clear evidence that the grouping strategy successfully steers the network toward a superior decision boundary. An analysis of the incorrectly predicted matches revealed that the MLP fails to account for the inherently deterministic and mutually exclusive nature of football match outcomes, whereby a Win for one team necessarily entails a Loss for the other.

Based on this, it is essential to consider the inherent determinism between the two opposing teams when using the model for prediction. Furthermore, coaches can enhance the team's performance-oriented results under limited training resources by transforming the high-impact technical statistics indicators identified by the model into training priorities, thereby achieving data-driven scientific training management.

## 6 Limitations

Although neural networks have been proven to be a powerful nonlinear machine learning method, in the context of football matches, where various chaotic events occur, even if there is a strong correlation between single-match statistical data and winning probability, it is possible that this relationship is mediated by other confounding factors or “random” effects. Furthermore, this study only included data from a single World Cup tournament, which has a relatively small sample size. These limitations may have resulted in some biases. Future research should consider objective bias between seasons while including data from more seasons to increase sample size.

At the same time, the main focus of this research is on some of TSI during the competition, while the objective environmental indicators such as (weather, humidity, venue, traffic appearance, temperature, diet, etc.) are not included in the consideration, and related studies have shown that these objective factors are also to a certain extent affecting the player's athletic performance status and thus affecting the model prediction. The findings of this study also suggest that future research should aim to incorporate multiple dimensions of influencing factors and incorporate as many objective variables and contextual factors as possible to provide a more comprehensive understanding of the relationship between statistical data and winning probability in football matches.

## Data Availability

Publicly available datasets were analyzed in this study. This data can be found here: https://www.fifa.com/en/match-centre/match/17/255711/285063/400235481.
