# Supplementary material for: Predicting football match outcomes: a multilayer perceptron neural network model based on technical statistics indicators of the FIFA world Cup
Source: Front Sports Act Living. 2025 Dec 3;7:1705198. doi: 10.3389/fspor.2025.1705198 (PMC12708546; doi:10.3389/fspor.2025.1705198)
Supplement: Supplementary file 1 [file Table1.docx]

**Predicting Football Match Outcomes: A** **Multilayer Perceptron Neural Network Model Based on** **Technical Statistics Indicators of the FIFA World Cup**

**Yingling Luo, Tao Quan*, Yongfeng Cao**

**College of Physical Education and Health Science, Chongqing Normal University, Chongqing, 401331, China**

**Supplementary Material**

Table 1 Tests for KMO and Bartlett

| KMO and Bartlett | |  |
| --- | --- | --- |
| Kaiser-Meyer-Olkin Measure of Sampling Adequacy | | 0.771 |
| Bartlett's Test of Sphericity | Approx. Chi-Square | 1772.314 |
|  | df | 231 |
|  | Sig. | <0.01 |
